# Dosimetric Comparison and Evaluation of Three Radiotherapy Techniques for Use after Modified Radical Mastectomy for Locally Advanced Left-sided Breast Cancer

**DOI:** 10.1038/srep12274

**Published:** 2015-07-21

**Authors:** Changchun Ma, Wuzhe Zhang, Jiayang Lu, Lili Wu, Fangcai Wu, Baotian Huang, Yan Lin, Dongsheng Li

**Affiliations:** 1 Department of Radiation Oncology, Cancer Hospital of Shantou University Medical College, Shantou, 515031, Guangdong Province, China; 2 Radiology Department, Second Affiliated Hospital, Shantou University Medical College, Shantou 515041, Guangdong Province, China

## Abstract

This study aimed to compare the post-modified radical mastectomy radiotherapy (PMRMRT) for left-sided breast cancer utilizing 3-dimensional conformal radiotherapy with field-in-field technique (3DCRT-FinF), 5-field intensity-modulated radiation therapy (5F-IMRT) and 2- partial arc volumetric modulated arc therapy (2P-VMAT). We created the 3 different PMRMRT plans for each of the ten consecutive patients. We performed Kruskal-Wallis analysis of variance (ANOVA) followed by the Dunn’s-type multiple comparisons to establish a hierarchy in terms of plan quality and dosimetric benefits. P < 0.05 was considered statistically significant. Both 5F-IMRT and 2P-VMAT plans exhibited similar PTV coverage (V_95%_), hotspot areas (V_110%_) and conformity (all p > 0.05), and significantly higher PTV coverage compared with 3DCRT-FinF (both p < 0.001). In addition, 5F-IMRT plans provided significantly less heart and left lung radiation exposure than 2P-VMAT (all p < 0.05). The 3DCRT-FinF plans with accurately estimated CTV displacement exhibited enhanced target coverage but worse organs at risk (OARs) sparing compared with those plans with underestimated displacements. Our results indicate that 5F-IMRT has dosimetrical advantages compared with the other two techniques in PMRMRT for left-sided breast cancer given its optimal balance between PTV coverage and OAR sparing (especially heart sparing). Individually quantifying and minimizing CTV displacement can significantly improve dosage distribution.

Breast cancer is the most common cancer among women worldwide. The mortality of breast cancer in developed countries has decreased since 1990, in part owing to effective screening and a combination of surgery, medicine and radiotherapy. Radiotherapy is an indispensable adjuvant treatment for patients undergoing breast-conserving surgery and for those with a high risk of recurrence after modified radical mastectomy^1^,^2^. However, in China, the morbidity and mortality resulting from breast cancer continue to rise. Modified radical mastectomy is still the most common treatment for breast cancer patients in China because of the prevalence of locally advanced breast cancer^3^, resulting from the lack of evidence-based early screening projects for breast cancer in this country. Breast conserving surgery for breast cancer is also limited by a lack of professional pathology support in basic units. Therefore, post modified radical mastectomy radiotherapy (PMRMRT) remains a major adjuvant treatment for breast cancer in China.

When irradiating the ipsilateral chest wall and supraclavicular region, PMRMRT for left-sided breast cancer inevitably leads to the irradiation of, the heart, ipsilateral lung and other organs at risk (OARs) with possible long-term adverse effects^4^,^5^. Adequate target dose coverage is a prerequisite for local control of breast cancer. However, dose inhomogeneity can influence the consistency of radiotherapy because the occurrence of dose hotspot areas frequently leads to severe acute radiation dermatitis (RD) during treatment^6^, and contribute to clinically significant late adverse effects^7^. Recently, an increasing number of reports have compared breast/chest wall volumetric modulated arc therapy (VMAT) or/and intensity-modulated radiation therapy (IMRT) and 3-dimensional conformal radiotherapy (3DCRT)^8^,^9^,^10^ . However, few studies have carried out a comprehensive dosimetric comparison and evaluation (including heart radiation exposure and plan evaluation, as well as the impact of CTV displacement on plan quality) of 3DCRT using field-in-field technique (3DCRT-FinF), 5-field IMRT (5F-IMRT) and optimized 2-partial arc VMAT (2P-VMAT) in post modified radical mastectomy radiotherapy (PMRMRT) plans for left-sided breast cancer patients. In our study, we compare and evaluate PMRMRT plans for patients with left-sided breast cancer utilizing 3DCRT-FinF, 5F-IMRT and 2P-VMAT techniques.

## Methods

Ten consecutive patients with left-sided, locally advanced breast cancer (median age 49 years, range 33 to 66 years) undergoing PMRMRT were enrolled in this study. Informed consent forms were signed by all patients. The study was performed in accordance with the Declaration of Helsinki, and was approved by the Ethics Committee of the Cancer Hospital of Shantou University Medical College. Patients were placed in a supine position. Planning images were acquired on a Philips Brilliance CT Big Bore Simulation System (Andover, MA) at a 5 mm slice thickness, as previously reported^11^. Clinical target volume (CTV) and OARs including heart, ipsilateral lung, left humeral head, spinal cord and contralateral breast and lung were contoured using the Eclipse treatment planning system (Eclipse 10.0, Varian Medical Systems, Palo Alto, CA, USA) based on Radiation Therapy Oncology Group (RTOG) Breast Cancer Contouring Atlas^12^. We expanded the CTV isotropically with a 0.7-cm margin in the chest wall section and a 0.5-cm border (subsequently retracted 0.3 cm from the surface of skin surface) in the supraclavicular section to generate the planning target volume (PTV). To manage the uncertain and low dose area of mega-voltage beams in the build-up region on the skin surface, so-called “skin flash”, we added a 1-cm thick tissue equivalent compensator to the surface of the chest walls.

### 3DCRT-FinF, 5F-IMRT and 2P-VMAT planning techniques

New plans using 3DCRT-FinF^9^,^13^,^14^, 5F-IMRT and 2P-VMAT for the 10 patients were created using an Eclipse treatment planning system. A Varian Truebeam linear accelerator (Varian Medical Systems, Palo Alto, CA, USA) with 6-MV photon energy and monoisocentric technique were used to simultaneously irradiate the chest wall and supraclavicular lymph nodes in all the plans. A prescription of 50 Gy in 2 Gy fractions to the PTV was used.

The 3DCRT-FinF plans consisted of two opposed open tangential half beams with gantry angles of approximately 300 and 120 degrees (no physical or dynamic wedges used), as well as one or two opposed half beam(s) for the supraclavicular field on the same side. Multileaf collimators (MLCs) were used to shield the heart, left lung and other organs at risk. Using one additional segment with the same angle as one of the tangential beams, a dose cloud was derived (individually for each patient) at a dose level of 107% from the optimized dose distribution of the 3-dimensional plan. The shape of this second segment was then conformed by means of a multileaf collimator to cover this dose cloud. Approximately 10% of the prescription dose was delivered with this shrunken field. If the 3DCRT-FinF plan did not achieve the optimized goals, two additional segments were used to conform to the 107% and 105% dose clouds successively, with each 5% of the prescription dose delivered with the corresponding shrunken field. To assess the influence of CTV displacement valuation on PTV coverage and OARs sparing, we created 2 sets of 3DCRT-FinF plans based on PTVs with CTV displacement estimation of 0.7 cm (3DCRT-FinF-PTV0.7 cm) and 0.5 cm (3DCRT-FinF-PTV0.5 cm), respectively.

The 5F-IMRT plans used 2 opposed tangential beams with the same gantry angles as those in 3DCRT-FinF, and 2 anterior beams with a 10-degree angle from the tangential ones, and a supraclavicular beam^8^,^9^,^10^,^15^. The plans were generated from a full inverse planning system. Each segment had 166 control points. The multileaf collimator moving speed was set at 2.5 cm per second. The doses were calculated using the anisotropic analytical algorithm (AAA, version 10.0.28) with a 2.5-mm grid, and optimized with dose volume optimizer (DVO) algorithm. The plans were delivered using sliding window technique with a dose rate of 600 MU/min.

The 2P-VMAT plans consisted of two optimized coplanar partial arcs, one with beam-on gantries rotating from 300 to 340 and from 80 to 120 degrees clockwisely, the other with the same beam-on gantries rotating counter-clockwise. Each arc was set with 98 control points. The 2P-VMAT plans were optimized using the progressive resolution optimizer 3 (PRO3) algorithm, based on the same constraints as the 5F-IMRT plans. The optimized goals, with priorities ranging from high to low, were as followings: PTV: D_95_ (95% of PTV receiving a prescription dose or higher) = 50Gy, V_47.5Gy_ ≥ 95%, V_53.5Gy_ ≤ 5%; heart: D_mean_ ≤ 10Gy, V_10Gy_ ≤ 20%, V_20Gy_ ≤ 15%^5^; left lung: D_mean_ ≤ 15Gy, V_10Gy_ ≤ 30%, V_20Gy_ ≤ 20%, V_30Gy_ ≤ 10%; right breast: D_max_ ≤ 3Gy; spinal cord: D_max_ ≤ 45Gy; left humeral head: D_mean_ ≤ 50Gy.

### Plan comparison and statistical analysis

V_95%_, V_110%_, heterogeneity index (HI) and conformity index (CI) values were calculated for the PTV. V_95%_ was defined as the percentage of the PTV receiving 95% or more of the prescription dose. V_110%_ indicated the dose hotspot area that received 110% of the prescription dose. The heterogeneity index was calculated as follows: HI = (D_2%_-D_98%_)/D_50%_, CI = (VPTV_ref_ /V_PTV_) × (VPTV_ref_ /V_ref_), where VPTV_ref_ represents the volume of PTV covered with the reference dose. V_PTV_ represents the volume of PTV and V_ref_ represents the volume covered with the reference dose or higher^16^. A higher HI value, ranging from 0 to 1, represents worse homogeneity. A higher CI value, ranging from 0 to 1, represents better conformity. D_2%_ represented the dose corresponding to 2% PTV volume as shown in the dose volume histogram (DVH) and could be deemed as the maximum dose, whereas D_98%_ could be deemed as the minimum dose. D_50%_ represented the reference dose (or prescription dose) for PTV.

For an overview of the dosimetry of the different techniques, we tested the parameters as follows^9^,^15^,^17^. D_mean_ is an average dose delivering to an organ. V_(xGy)_ represents the percentage of an organ’s volume receiving (x) Gy or higher. D_mean_, V_5Gy_, V_10Gy_, V_20Gy_ and V_30Gy_ were calculated for the heart, and D_mean_, V_5Gy_, V_10Gy_ and V_20Gy_ were calculated for the left lung. D_mean_ was assessed for the contralateral breast and ipsilateral humeral head separately. V_45Gy_ was calculated for spinal cord, and V_50Gy_ was calculated for healthy tissue.

We performed Kruskal-Wallis analysis of variance (ANOVA) followed by Dunn’s-type multiple comparisons between any two of the three techniques, to establish a hierarchy in terms of plan quality and dosimetric benefits. We used paired-sample *t*-tests to compare the normally distributed data between the 3DCRT-FinF plans with a presumably accurate estimation of CTV displacement (PTV 0.7 cm) and those with underestimation (PTV 0.5 cm). P values less than 0.05 were considered statistically significant. The SPSS 19.0 software (IBM, Chicago, IL) was used for statistical data management and analysis.

## Results

### Target coverage

**Figure 1:**
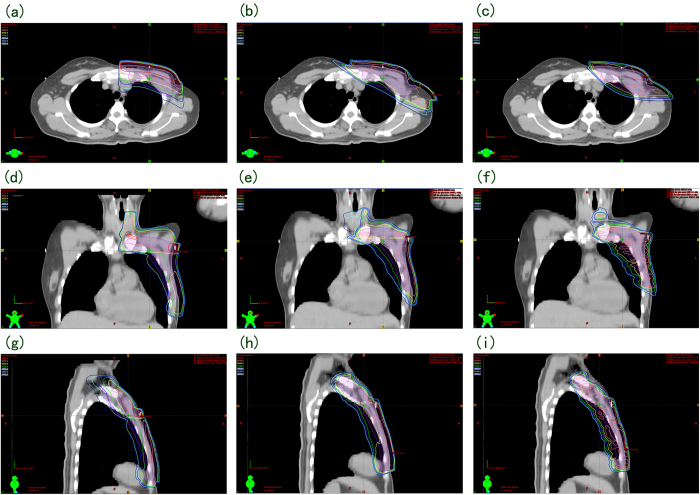
Transverse, coronal and sagittal dose distribution curves for the 3 techniques in a representative patient. (**a, d, g**), (**b, e, h**) and (**c, f, i**) showed the dosage distribution for 3DCRT-FinF, 5F-IMRT and 2P-VMAT, respectively.

**Figure 2:**
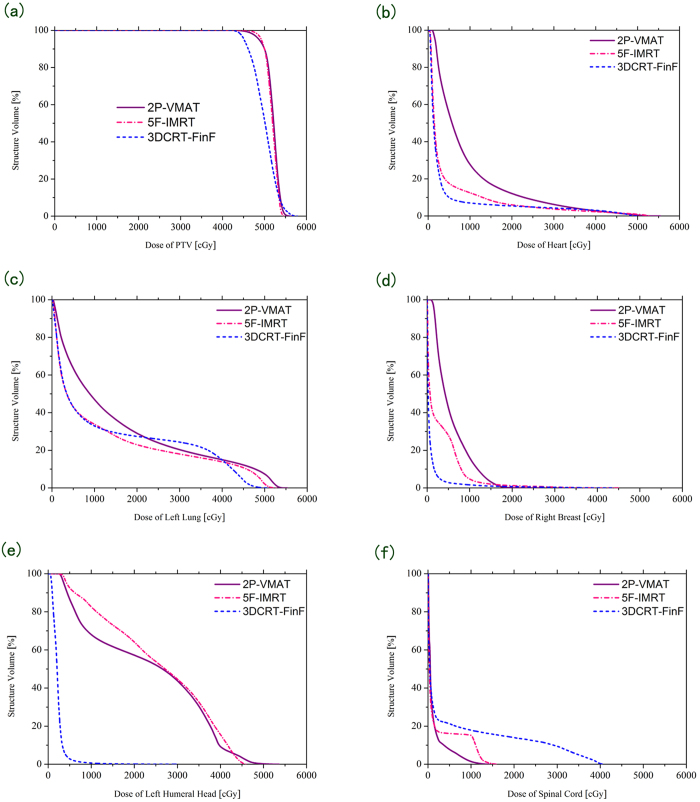
Comparison of dose volume histograms (DVHs) among the new 3DCRT-FinF, 5F-IMRT and 2P-VMAT plans. The charts show the DVHs for PTV (**a**), heart (**b**), left lung (**c**), right breast (**d**), left humeral head (**e**) and spinal cord (**f**).

The mean volumes, averaged from the 10 patients, for PTV, heart, left lung, right lung, right breast and left humeral head were 813.16 ± 176.78, 486.54 ± 91.45, 982.23 ± 205.19, 1195.87 ± 221.02, 458.99 ± 232.25 and 41.13 ± 7.94 (cm^3^), respectively. There were significant differences in D_2%_, D_98%_, V_95%_, V_110%_, HI and CI in PTV among the new 3DCRT-FinF, 5F-IMRT and 2P-VMAT plans (Fig. 1, Fig. 2 and Table 1). For example, the 5F-IMRT (V_95%_ = 99.16 ± 0.33) and 2P-VMAT (V_95%_ = 98.45 ± 0.51) plans provided significantly increased PTV dose coverage compared with the 3DCRT-FinF plans (V_95%_ = 78.23 ± 4.25) (both p < 0.001). Both 5F-IMRT and 2P-VMAT plans exhibited similar PTV dose coverage, hotspot areas (V_110%_), homogeneity and conformity, with V_95%_ values of 99.16 ± 0.33 and 98.45 ± 0.51, V_110%_ values of 0.22 ± 0.43 and 2.09 ± 3.38, HI values of 0.107 ± 0.013 and 0.124 ± 0.025, and CI values of 0.64 ± 0.07 and 0.68 ± 0.07, respectively (all p > 0.05) (Fig. 1 and Table 1). The averages total MUs were 456.10 ± 20.98, 1021.10 ± 343.10 and 403.60 ± 31.60 MU for the 3DCRT-FinF, 5F-IMRT and 2P-VMAT plans, respectively (Table 1).

### Radiation exposure of OARs and other healthy tissues

Among the 3DCRT-FinF, 5F-IMRT and 2P-VMAT plans, the following significant differences were noted: D_mean_, V_5Gy_, V_10Gy_ and V_20Gy_ in the heart; D_mean_, V_5Gy_ and V_10Gy_ in the left lung; D_mean_ and V_5Gy_ in the right breast; and D_mean_ in the left humeral head (Fig. 2 and Table 2). There were no statistically significant differences among the aforementioned 3 techniques for V_30Gy_ in the heart, V_20Gy_ in the left lung, V_45Gy_ in the spinal cord and V_50Gy_ in the healthy tissue (Fig. 2 and Table 2). In addition, the 5F-IMRT and 3DCRT-FinF plans exhibited similar D_mean_ values (8.08 ± 2.73 vs. 7.29 ± 3.05 Gy, p = 0.887), V_5Gy_ values (28.66 ± 10.39 vs. 22.14 ± 8.64, p = 0.276), V_10Gy_ values (17.85 ± 7.09 vs. 14.95 ± 6.74, p = 0.739) and V_20Gy_ values (10.54 ± 5.17 vs. 12.48 ± 6.36, p = 0.808) in the heart as well as similar D_mean_ values (15.03 ± 3.09 vs. 15.32 ± 2.72 Gy, p = 0.975), V_5Gy_ values (52.53 ± 7.65 vs. 49.63 ± 7.76, p = 0.707) and V_10Gy_ values (36.89 ± 7.75 vs. 37.52 ± 7.10, p = 0.983) in the left lung. For the 5F-IMRT plans, V_5Gy,_ V_10Gy_ and V_20Gy_ in the heart as well as D_mean_, V_5Gy_ and V_10Gy_ in the left lung were correspondingly significantly lower than in 2P-VMAT (all p < 0.05) (Table 2).

### Valuation of the CTV displacement significantly influences target coverage and OAR sparing

**Figure 3:**
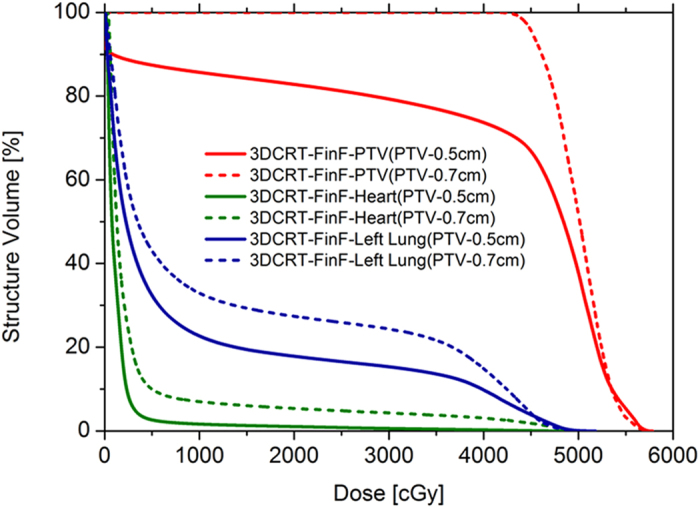
Comparison of DVHs between 3DCRT-FinF plans with a PTV of 0.5 cm and a PTV of 0.7cm. The two sets of 3DCRT-FinF plans for each patient were created based on CTV displacement estimations of 0.7 cm and 0.5 cm, respectively. The comparison was performed presuming that the “actual” displacement of the CTVs was 0.7 cm isotropically.

The 3DCRT-FinF-PTV 0.7 cm plans based on accurately estimated CTV displacement values of 0.7 cm exhibited better PTV coverage but worse OAR (heart, left lung) sparing compared with the 3DCRT-FinF-PTV 0.5 cm plans based on underestimation of CTV displacement values of 0.5 cm, assuming that the “actual” displacement of CTV was 0.7 cm isotropically (Fig. 3 and Table 3).

## Discussion

The management of invasive breast cancer has changed substantially over the past few decades. A larger proportion of such patients, especially in developed nations, is now treated with breast-conserving surgery rather than mastectomy, with increasing numbers of patients receiving systemic therapy^18^. However, PMRMRT remains a major adjuvant treatment for women in China because locally advanced breast cancer and corresponding use of modified radical mastectomy are presently common in China. Two different postoperative radiotherapy techniques are used for patients after breast-conserving surgery and radical mastectomy. Patients undergoing breast-conserving surgery are at an earlier stages and need only whole breast irradiation, whereas patients with locally advanced stages typically require modified radical mastectomy and irradiation of the ipsilateral chest wall and supraclavicular region. In our study, to manage the dose uncertainty of mega-voltage beams in the build-up region^19^,^20^, as well as the CTV displacement resulting from intrafraction movements and setup errors^21^, we added a 1.0-cm thick tissue equivalent compensator to the surface of the chest walls in order to cover the farthest CTV displacement border. A “skin flash” could be avoided because a tissue equivalent compensator that was at least 0.3 cm thick was maintained on the surface of the chest wall despite the CTV displacement.

We found that 5F-IMRT plans exhibited advantageous dosimetry compared with the 3DCRT-FinF and 2P-VMAT plans in PMRMRT for left-sided breast cancer. The PTV coverage, dose homogeneity and conformity of IMRT were enhanced compared with the 3DCRT-FinF plans. Moreover, the 5F-IMRT plans exhibited better heart and ipsilateral lung sparing compared with the 2P-VMAT plans. However, whether the dosimetric advantages of 5F-IMRT leading to significant clinical benefit remains unclear and warrants further study. The IMRT plans had an average MU number per fraction equal to 1021 (corresponding to 10.21Gy), which was more than 5 times the number for a 2 Gy/fraction prescription. These plans would require longer periods for treatment and for pre-treatment dosimetric verification compared with the 3DCRT-FinF and 2P-VMAT plans. However, the 5F-IMRT plans were delivered with a sliding window technique at a dose rate of 600 MU/min, using a Varian Truebeam linear accelerator. These plans would require a beam-on time of approximately 2 min, suggesting that it is clinically applicable.

Potential long-term sequelae of post mastectomy radiotherapy include cardiac toxicity^5^, radiation pneumonitis^22^, lymphedema^23^, rib fractures^24^, brachial plexopathy^25^, and radiation-induced second malignancy^26^. These toxicities, except for second neoplasms resulting from a stochastic effect, generally result from the deterministic effects of irradiation, which have been reduced to an acceptable level with the utilization of modern radiotherapy techniques such as 3DCRT-FinF. Modern techniques have also decreased the exposure of the heart to radiation^27^, however, the heart still receives 1 to 5 Gy irradiation in most patients. Studies have demonstrated that this exposure level can lead to ischaemic heart disease^28^. In a population-based study of the incidence of major ischaemic cardiac events in women who had received radiation therapy for breast cancer, Darby and colleagues reported that the rate of ischaemic cardiac disease increased with radiation exposure and that each Gy of radiation was associated with a 7.4% increase in the risk of a subsequent major coronary event, regardless of the minimum dose^5^. The risk of major coronary events increases within 5 years and continues for at least 2 decades after radiation exposure. This increased risk of a major coronary event also applies to radiation technologies used after 1990. The absolute radiation-related risk of a major coronary event also increases significantly in breast cancer patients with ischaemic cardiac disease or preexisting cardiac risk factors. Therefore, current radiotherapy techniques are insufficient to eliminate the possibility of delayed cardiac toxicity. Heart irradiation is closely associated with the development of life-threatening major cardiac events, including myocardial infarction, coronary revascularization, or death from ischaemic cardiac disease. Based on the characteristics of radiation-related major coronary events, including the lack of a threshold value, their long-term nature, the dosage-related effect and the additive nature of the risk with preexisting cardiac diseases, we should be concerned about both disease (breast cancer)-specific long-term survival and the influence of radiation-related coronary events on long-term survival and quality of life of patients with left-sided breast cancer at the beginning of their treatment. Therefore, we believe that the exposure of the heart to radiation in thoracic radiotherapy, especially in patients with long life expectancies, must be assessed and limited more accurately and stringently. Exposure could be used as an a priori limitation parameter to evaluate which of several PMRMRT plans for left-sided breast cancer is more advantageous if PTV dose coverage and other OAR sparings are acceptable. In our study, both 5F-IMRT and 2P-VMAT plans achieved perfect homogenous and conformal PTV coverage, which might improve local tumor control and reduce hotspot area-related toxicity^6^,^7^. The reduction in heart and left lung radiation exposure in 5F-IMRT plans compared with 2P-VMAT plans resulted from the narrower included angle (10 degrees) between the tangential beam and the adjacent anterior beam in 5F-IMRT plans. The angles of starting and ending beams in 2P-VMAT were the same as those of the corresponding tangential beams in 5F-IMRT. The included angles (40 degrees) between the starting gantry and the adjacent blocking end of the arc were finalized after adjustment of the blocking end for every 5 degrees, ranging from 5 to 90 degrees, to achieve the best balance of PTV coverage and heart and left lung sparing. The 2P-VMAT plans achieved an average heart D_mean_ of 11.9 ± 5 Gy which was consistent with reported values (between 11.4 to 12.9 Gy)^17^,^29^,^30^. The average values of D_mean_. and V_20Gy_ for the left lung were 18.6 Gy and 34.1% respectively. Whether these values could be clinically acceptable needs to be verified, although a value of V_20Gy_ ≥ 25% for the whole lung could be a predictive factor for symptomatic pneumonitis in radiotherapy for lung cancer^31^,^32^. Therefore, despite having similarly perfect PTV coverage, hotspot area (V_110%_) and CI to those of 5F-IMRT plans, or even requiring much shorter treatment times, we still believed that 2P-VMAT plans should not be the preferred option for PMRMRT for left-sided breast cancer owing to its significantly increased heart and left lung exposure compared with 5F-IMRT.

Precise radiotherapy techniques, including IMRT and VMAT, have been clinically implemented in head and neck tumors, such as nasopharyngeal carcinoma^33^. However, the utilization of such precise technology in thoracic targets inevitably creates the problems of set up and respiration motion uncertainties. Our data also indicate that the estimation of CTV displacement significantly influences target coverage and OAR sparing (Fig. 3 and Table 3), suggesting that individually quantifying and minimizing CTV displacement for left-sided breast cancer PMRMRT plans can significantly improve dosage distribution. The displacement of CTV in the chest wall is mainly caused by respiration because respiratory motion leads directly to chest wall movement and deviations in the patient’s position. In addition to intrinsic systemic errors, deviations in the patients’ position mainly arise from inconsistent respiration phases between simulation and treatment. Therefore, an isotropic 0.7- cm expanding margin of CTV was referred to as an “approximate value” in a published study^11^ and should not serve as the standard of PTV expansion for all patients. The CTV displacement could differ significantly among women of different races, among different individuals within the same race, among different phases of a respiratory cycle and among three different dimensional directions within the same patient. Moreover, such differences cannot be clinically neglected. The PTV expansion can be tailored by quantifying respiration-induced CTV displacement on a patient-by-patient basis via 4-dimensional computed tomography (4DCT) simulation scanning and by maintaining the consistency of breathing-coupled position alterations between simulation and treatment. Maximum intensity projection (MIP)-reconstructed 4DCT, breath holding and respiration-gating techniques^21^,^34^ provide possible ways to implement this procedure.

In conclusion, 5F-IMRT is more dosimetrically advantageous in PMRMRT for left-sided breast cancer patients owing to its enhanced PTV coverage and similar heart and left lung sparing properties compared with 3DCRT-FinF as well as enhanced heart and left lung sparing and similar PTV coverage compared with 2P-VMAT. However, the shortcomings of this technique include a high MU number, a long treatment time, and a need for pre-treatment dosimetric verification. Individually quantifying and minimizing PTV for left-sided breast cancer PMRMRT plans can significantly improve dosage distribution. We can further minimize and tailor the PTV expansion by quantifying respiration-induced CTV displacement on a patient-by-patient basis through 4DCT simulation scanning and maintaining the consistency of breathing-coupled position alteration between simulation and treatment.

## Additional Information

**How to cite this article**: Ma, C. *et al*. Dosimetric Comparison and Evaluation of Three Radiotherapy Techniques for Use after Modified Radical Mastectomy for Locally Advanced Left-sided Breast Cancer. *Sci. Rep*. **5**, 12274; doi: 10.1038/srep12274 (2015).

## Figures and Tables

**Table 1:** PTV coverage based on DVH analysis.

Parameters	3DCRT-FinF	5F-IMRT	2P-VMAT	F	p1	p2	p3
D_2%_	55.50 ± 0.71	54.00 ± 0.51	54.93 ± 0.87	11.20	< 0.001	< 0.194	0.019
D_98%_	44.18 ± 0.56	48.29 ± 0.30	47.77 ± 0.35	285.72	< 0.001	< 0.001	0.026
V_95%_	78.23 ± 4.25	99.16 ± 0.33	98.45 ± 0.51	229.81	< 0.001	< 0.001	0.797
V_110%_	4.26 ± 3.73	0.22 ± 0.43	2.09 ± 3.38	4.81	0.022	0.466	0.309
HI	0.235 ± 0.017	0.114 ± 0.012	0.143 ± 0.025	122.63	< 0.001	< 0.001	0.002
CI	0.27 ± 0.07	0.64 ± 0.07	0.68 ± 0.07	102.79	< 0.001	< 0.001	0.425
MU	456.10 ± 20.98	1021.10 ± 343.10	403.60 ± 31.60	29.51	0.002	0.001	0.001

Abbreviations: Vx = volume (%) receiving x dose (Gy) or higher; 3DCRT-FinF = three-dimensional conformal radiotherapy using

field-in-field technique; 5F-IMRT = 5-field intensity-modulated radiotherapy; 2P-VMAT = 2-partial arc volumetric modulated arc therapy.

Data presented as mean ± standard deviation. F values from ANOVA analysis (α = 0.05). p1: 3DCRT & 5F-IMRT;

p2: 3DCRT-FinF & 2P-VMAT; p3: 5F-IMRT & 2P-VMAT. D_2%_ = the maximum dose; D_98%_ = the minimum dose.

**Table 2:** Radiation exposure of heart and other normal tissues.

Organ at risk	Parameters	3DCRT-FinF	5F-IMRT	2P-VMAT	F	p1	p2	p3
**Heart**	**D**_**mean**_	7.29 ± 3.00	8.08 ± 2.73	11.90 ± 5.06	4.25	0.887	0.029	0.079
	**V**_**5Gy**_	22.14 ± 8.64	28.66 ± 10.39	68.14 ± 8.73	71.82	0.276	< 0.001	< 0.001
	**V**_**10Gy**_	14.95 ± 6.74	17.85 ± 7.09	42.33 ± 11.42	30.02	0.739	< 0.001	< 0.001
	**V**_**20Gy**_	12.48 ± 6.36	10.54 ± 5.17	19.48 ± 8.84	4.56	0.808	0.081	0.021
	**V**_**30Gy**_	10.74 ± 5.89	7.86 ± 4.53	11.14 ± 6.19	1.03	0.490	0.986.	0.399
**Left lung**	**D**_**mean**_	15.32 ± 2.72	15.03 ± 3.09	18.57 ± 3.17	4.30	0.975	0.056	0.035
	**V**_**5Gy**_	49.63 ± 7.76	52.53 ± 7.65	70.36 ± 8.84	19.19	0.707	< 0.001	< 0.001
	**V**_**10Gy**_	37.52 ± 7.10	36.89 ± 7.75	51.67 ± 8.72	11.22	0.983	0.001	0.001
	**V**_**20Gy**_	31.36 ± 6.04	27.77 ± 7.08	34.08 ± 7.16	2.18	0.473	0.647	0.113
**Right breast**	**D**_**mean**_	1.68 ± 1.60	2.81 ± 2.23	5.79 ± 2.71	9.09	0.504	0.001	0.016
**Left humeral head**	**D**_**mean**_	12.65 ± 10.40	33.54 ± 9.00	40.77 ± 10.74	21.01	< 0.001	< 0.001	0.261
**Spinal cord**	**V**_**45Gy**_	2.46 ± 4.23	0	0	—	—	—	—
**Healthy tissue**	**V**_**50Gy**_	2.45 ± 2.38	1.55 ± 0.71	1.48 ± 0.76	1.29	0.386	0.336	0.995

Abbreviations: Dmean = mean dose (Gy); Vx = volume (%) receiving x dose (Gy) or higher; 3DCRT-FinF = three-dimensional conformal radiotherapy using field-in-field technique; 5F-IMRT = 5-field intensity-modulated radiotherapy; 2P-VMAT = 2 partial arcs volumetric modulated arc therapy. Data presented as mean ± standard deviation. F values from ANOVA analysis(α = 0.05). p1: 3DCRT-FinF & 5F-IMRT; p2: 3DCRT-FinF & 2P-VMAT; p3: 5F-IMRT & 2P-VMAT.

**Table 3:** The influence of CTV displacement estimation on PTV coverage and OAR sparing.

Organ at risk	Parameters	3DCRT-FinF -PTV0.7 cm	3DCRT-FinF -PTV0.5 cm	p
**PTV**	**V**_**95%**_	78.23 ± 4.25	56.94 ± 5.62	< 0.001
**Heart**	**D**_**mean**_	7.29 ± 3.05	3.80 ± 2.20	< 0.001
	**V**_**5Gy**_	22.14 ± 8.64	9.40 ± 6.47	< 0.001
	**V**_**10Gy**_	14.95 ± 6.74	6.83 ± 5.21	< 0.001
	**V**_**20Gy**_	12.48 ± 6.36	5.36 ± 4.44	< 0.001
	**V**_**30Gy**_	10.74 ± 5.89	4.24 ± 3.68	< 0.001
**Left lung**	**D**_**mean**_	15.32 ± 2.72	10.91 ± 2.75	< 0.001
	**V**_**5Gy**_	49.63 ± 7.76	35.97 ± 8.40	< 0.001
	**V**_**10Gy**_	37.52 ± 7.10	25.63 ± 6.95	< 0.001

p:3DCRT-FinF-PTV0.7 cm (3DCRT-FinF plans generated from PTV0.7 cm with presumably accurate estimation of CTV displacement of 0.7 cm) & 3DCRT-FinF-PTV0.5 cm (3DCRT-FinF plans generated from PTV0.5 cm with underestimation of CTV displacement, presuming that the actual CTV displacement valuation was 0.7 cm). Data presented as mean ± standard deviation. p-values from paired-sample *t* test.
